# Photostability of Perovskite Solar Cells: Challenges and Strategies

**DOI:** 10.3390/nano15110786

**Published:** 2025-05-23

**Authors:** Ruohan Liu, Runnan Yu, Zhan’ao Tan

**Affiliations:** Beijing Advanced Innovation Center for Soft Matter Science and Engineering, College of Materials Science and Engineering, Beijing University of Chemical Technology, Beijing 100029, China; lrh619588@163.com (R.L.); tanzhanao@mail.buct.edu.cn (Z.T.)

**Keywords:** perovskite solar cells, photostability, UV resistance, device engineering

## Abstract

Perovskite solar cells (PSCs) have been regarded as a revolutionary technology in the photovoltaic field, offering a promising pathway for efficient and cost-effective solar energy conversion and demonstrating broad prospects for future green energy technologies. However, critical stability challenges, specifically degradation induced by humidity, light, or heat, severely hinder the commercialization of this technology. Specifically, ultraviolet (UV) radiation in the solar spectrum is a major factor leading to the degradation of perovskite materials. This review focuses on the challenges and strategies for addressing the photostability issues of PSCs. A variety of strategies have been explored, which can be classified as external protection (such as UV-blocking encapsulation technologies) and internal optimization approaches (including precise compositional tuning, the incorporation of functional additives, interface engineering, and improvements to charge transport layers). Finally, this review delves into the key scientific challenges and technological bottlenecks currently faced in addressing the UV stability of PSCs and proposes future directions for solving UV stability issues. It also provides an outlook on the future development prospects of these technologies.

## 1. Introduction

Against the backdrop of the global energy transition and the pursuit of carbon neutrality goals, solar energy has been widely recognized as a key renewable energy source due to its abundant availability, as well as its clean and sustainable nature [1,2,3]. Compared with other energy sources, the development and utilization of solar energy cause minimal negative environmental impacts, making it an important option for achieving sustainable development goals [4]. Among various solar energy utilization technologies, photovoltaic (PV) power generation has attracted extensive attention from both academia and industry due to its high energy conversion efficiency and environmental friendliness [5]. The core principle of this technology is based on the photovoltaic effect, whereby solar cells made of semiconductor materials directly convert photon energy from solar radiation into electricity [6]. Metal halide perovskites, owing to their long carrier diffusion lengths, high absorption coefficients, high radiative recombination rates, and defect tolerance, are considered excellent candidates for next-generation optoelectronic applications, including solar cells, LEDs, and photodetectors [7]. Perovskite solar cells (PSCs), with their high energy conversion efficiency and low fabrication cost, have become one of the most promising solar energy utilization technologies [8]. To date, the power conversion efficiency (PCE) of single-junction perovskite solar cells has reached 26.9% [9]. As widely recognized in the research community, water molecules (H_2_O) and oxygen (O_2_) are the most direct environmental factors leading to the degradation of perovskite materials [10]. At the molecular level, water molecules undergo hydrolysis reactions with the organic components in perovskites (such as methylammonium ions) and promote the migration and loss of halide ions. Oxygen, on the other hand, reacts with lead sites in perovskite to form oxidation products. These processes irreversibly damage the perovskite crystal structure, leading to the significant deterioration of its optoelectronic properties [11]. However, as photovoltaic devices rely on sunlight, photo-induced degradation also presents a significant challenge [12,13,14]. Prolonged illumination not only induces phase segregation and ion migration in perovskite materials, but also accelerates the aforementioned water and oxygen corrosion processes. Under ultraviolet (UV) illumination in particular, high-energy photons directly break chemical bonds in the perovskite, generating numerous defect states. More complexly, the coupled effects of light, heat, and electric fields synergistically accelerate device performance degradation, making the issue of stability in PSCs particularly prominent under real-world operating conditions. The inherent instability under UV illumination severely limits their practical applications [15]. The UV light in sunlight can excite photogenerated carriers in PSCs, contributing partially to the photocurrent; however, this high-energy UV radiation negatively impacts the stability of PSCs, significantly shortening their lifetime under prolonged UV exposure [16,17,18]. Therefore, the poor photostability of PSCs remains a critical issue that needs to be urgently addressed. However, related studies and reviews rarely mention or systematically summarize strategies to address the photostability and ultraviolet stability of PSCs, leading to the frequent neglect of UV-induced device damage.

In this review, we systematically explore the mechanisms by which light and UV radiation impact the performance and stability of PSCs, and comprehensively summarize recent advances in improving PSC photostability. According to their modes of action, these strategies for enhancing stability can be categorized into two major types: external and internal approaches. External strategies mainly involve physical protection methods such as device encapsulation optimization, UV-filtering layer design, and environmental control. Internal strategies focus on the intrinsic modification of material systems, specifically including perovskite compositional engineering, additive engineering, interface engineering, and defect passivation strategies. By analyzing the underlying mechanisms and practical effectiveness of these strategies, this review aims to provide systematic research directions and feasible technical pathways to fundamentally address the photostability issues of PSCs, thereby promoting the commercialization of perovskite photovoltaic technologies.

## 2. Photostability of PSCs

### 2.1. Ultraviolet Irradiation

Solar radiation can be divided into three major regions based on wavelength: the infrared region, the visible region, and the ultraviolet (UV) region [19]. First, radiation with wavelengths greater than 760 nm is referred to as the infrared region, characterized primarily by thermal effects. Second, radiation within the wavelength range of 380–760 nm constitutes the visible region. Finally, radiation within the wavelength range of 10–380 nm falls into the UV region [20], which can be further subdivided into two major subregions. The first subregion of the UV spectrum is the far-ultraviolet (FUV) region, with wavelengths ranging from 10 to 200 nm [21]. Radiation in this wavelength range is strongly absorbed by molecules in the Earth’s atmosphere, such as nitrogen, oxygen, carbon dioxide, and water vapor. Due to this strong absorption, FUV radiation cannot propagate under normal atmospheric conditions and can only exist in a vacuum environment; hence, it is specifically referred to as “vacuum ultraviolet” (VUV) radiation [22]. As a result, FUV radiation is virtually absent at the Earth’s surface and can only be observed using specialized vacuum experimental equipment. The second subregion of the UV spectrum is the near-ultraviolet (NUV) region, with wavelengths ranging from 200 to 380 nm. Unlike FUV radiation, NUV radiation can penetrate the atmosphere and reach the Earth’s surface. Based on differences in wavelength range and physical properties, the NUV region can be further subdivided into three sub-bands: UVC, UVB, and UVA [23]. The first sub-band is UVC, covering wavelengths from 200 to 280 nm. UV radiation in this band has very weak penetration ability and cannot pass through most transparent glass and plastic materials. Fortunately, UVC radiation from the sun is almost completely absorbed by the Earth’s ozone layer, and thus rarely reaches the Earth’s surface. The second sub-band is UVB, with wavelengths ranging from 280 to 320 nm. UV radiation in this range exhibits moderate penetration ability, with the shorter wavelengths being absorbed by transparent glass. In solar radiation, most UVB radiation is absorbed by the ozone layer, with less than 2% reaching the Earth’s surface. The intensity of UVB radiation varies with seasons and time of day, being particularly strong during summer and at noon. The third sub-band is UVA, with wavelengths ranging from 320 to 380 nm. UV radiation in this band has a strong penetration ability and can pass through most transparent glass and plastic materials [24]. In solar radiation, more than 98% of UVA radiation penetrates the ozone layer and clouds to reach the Earth’s surface, making it the most prevalent type of UV radiation encountered by PSCs in daily operations [25,26,27].

UV light consistently causes significant damage to PSCs, relentlessly degrading the perovskite material and shortening the operational lifetime of devices [28,29,30,31]. Under sunlight exposure, including both UVA (320–380 nm) and UVB (280–320 nm) radiation, perovskite decomposition cannot be effectively suppressed. Intense UVA and UVB radiation readily damage Pb-based perovskite devices, causing the reduction of Pb^2+^ to metallic Pb^0^, which leads to the formation of primary deep defect states and severely deteriorates the performance and stability of PSCs [32].

### 2.2. Degradation Mechanisms and Contributing Factors

#### 2.2.1. Ultraviolet-Induced Degradation Mechanism

The instability of PSCs is closely related to the presence of iodide ions [33]. The core mechanism underlying the ultraviolet-induced degradation of perovskites can be summarized into the following key processes: (i) Initial photolysis reaction: High-energy UV photons (hν > 2.5 eV) directly break the Pb-I bonds within the perovskite, resulting in lattice disruption [34]. This process dynamically generates three types of defects: iodide vacancies (V_I_), which are electrically neutral yet serve as active sites for subsequent degradation; interstitial iodide (I′), which provides channels for ion migration; and molecular iodine (I_2_), a key intermediate that triggers chain reactions [35]. (ii) Self-accelerating degradation cycle: The generated I_2_ molecules dissociate under illumination into highly reactive iodine radicals (I·), which subsequently attack the organic cations (MA^+^/FA^+^), inducing dehydrogenation reactions that produce additional I_2_ molecules in a positive feedback loop, thereby accelerating the decomposition of the perovskite lattice (e.g., converting it into PbI_2_) [36]. (iii) Ion migration and interfacial corrosion: Under the combined influence of light and an electric field, I^−^ ions can migrate with activation energies as low as 0.2 eV/μm [37]. The accumulation of these migrating ions at the electrode interfaces triggers metal corrosion, leading to the formation of an irreversible interfacial barrier layer. The generation of I_2_ implies the formation of iodide vacancy defects within the crystal structure, which further accelerates the degradation of the perovskite [38,39,40]. The presence of metallic lead (Pb^0^) as an interfacial trap site directly leads to nonradiative carrier recombination, severely impairing the photovoltaic performance and stability of PSCs [41,42,43].

These processes mutually reinforce one another to form a synergistic degradation network—termed the “photolysis–migration–corrosion” cascade—where photolysis generates migrating species (I^−^), migration leads to interfacial degradation, and the resulting corrosion products further accelerate bulk decomposition. This multi-stage chain reaction is the fundamental cause of the rapid failure of perovskite devices under UV illumination [44,45].

#### 2.2.2. Defects

UV radiation can induce oxygen vacancies and defects in perovskite crystals, leading to device breakdown. Under illumination, the presence of defects within the perovskite films and at the interfaces further accelerates the degradation process; these defects act as nonradiative charge recombination centers, thereby reducing device efficiency. FAPbI_3_ degrades from the photoactive cubic α-FAPbI_3_ perovskite phase to the hexagonal non-perovskite δ-FAPbI_3_ phase, while MAPbI_3_ degrades into PbI_2_. UV radiation under sunlight may induce deep defect states (e.g., reduction of Pb^2+^ to Pb^0^) [46,47,48], thereby severely compromising the performance and stability of PSCs [49,50]. Hexagonal perovskites have been shown to undergo localized photodegradation under operational conditions [51], and studies reveal that hexagonal polymorphic defect clusters are among the most severe types of carrier trapping centers [52]. They also act as intermediates during perovskite crystallization and degradation processes [53].

#### 2.2.3. Ion Migration

**Figure 1:**
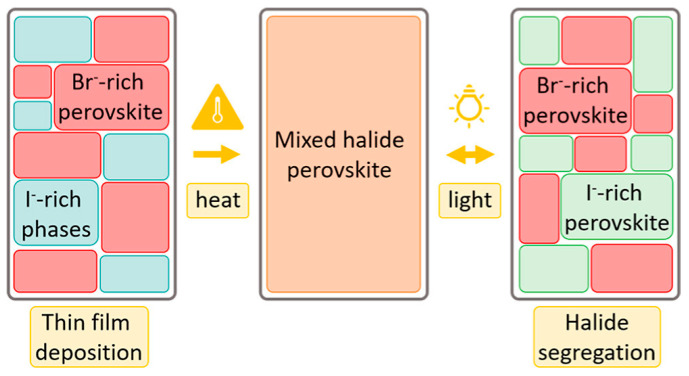
Mixed-halide perovskites exhibit a random and uniform distribution of halide ions in the dark, while phase-separating into Br-rich and I-rich domains under illumination. Reproduced with permission from [57], Copyright 2024, American Chemical Society.

Ion migration occurs within the perovskite films. Ion migration is a potential source of instability, highlighting the importance of further elucidating this process to develop strategies for improving photostability [54]. Uniformly mixed halide perovskites (e.g., APb(I_1−x_Br_x_)_3_) can reversibly segregate under continuous visible-light irradiation into narrow-bandgap I-rich domains and wide-bandgap Br-rich domains (Figure 1). This light-induced halide segregation is essentially a result of ion migration. Iodine-rich domains reduce the local open-circuit voltage (*V*_OC_) and lower the power conversion efficiency of mixed-halide perovskite solar cells [55]. For phase-unstable wide-bandgap perovskite films, when more than 20% of bromide (Br) is substituted by iodide (I) to widen the bandgap, phase separation under illumination readily occurs, forming Br-rich domains with higher bandgaps and I-rich domains with lower bandgaps. In such cases, the iodide-rich regions exacerbate the open-circuit voltage (*V*_OC_) deficit and compromise device stability [56].

## 3. Strategies for Enhancing Photostability

### 3.1. External Strategies

One of the most straightforward and easily implementable approaches to address the photostability issues of PSCs is to introduce a UV filtering layer on the glass side (incident light side) of the device [58]. This method physically blocks high-energy UV photons from directly entering the device, thereby preventing UV-induced degradation reactions at the source. A key advantage of this approach is its strong process compatibility, allowing direct integration into existing module encapsulation processes without altering the device structure. It is also low in cost—significantly lower than other stabilization strategies.

**Figure 2:**
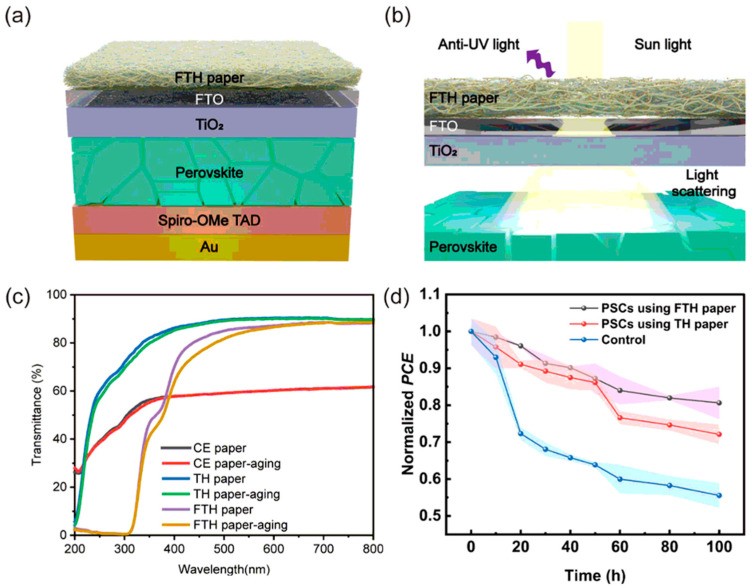
(**a**) Schematic illustration of FTH paper adhered to the FTO glass surface of PSCs. (**b**) Schematic diagram of the light-scattering mechanism of FTH paper in PSCs. (**c**) Comparison of the transparency of CE paper (control group), TH paper (non-UV resistant), and FTH paper (UV resistant) before and after 100 h of UV aging. (**d**) PCE degradation curves under UV illumination. Reproduced with permission from [59], Copyright 2024, Springer Nature.

Si et al. developed a functional cellulose paper (FTH paper) with high transparency, high haze, and UV-blocking capability, suitable for industrial production as a paper-based coating for PSCs (Figure 2). It can be attached to FTO or ITO glass surfaces using a 10 wt% PVP solution, effectively extending the operational lifetime of PSCs and significantly enhancing their optical path length and UV stability (with a 26% improvement after 100 h) [59]. When the impregnation amounts of carboxymethyl cellulose and tannic acid were 16 wt% and 0.7 wt%, respectively, the transmittance and UV-blocking efficiency reached 86.8% (at 600 nm) and 83.1% (at 320 nm), respectively, while maintaining a haze of 71.5%. After protonation and desalination treatments, the FTH paper exhibited excellent water resistance and mechanical properties (71.49 MPa tensile strength, 2156 folding cycles) [59].

Although complete UV filtering can significantly enhance the stability of PSCs, it inevitably reduces the photocurrent and PCE of the devices. Studies have shown that completely blocking UV radiation (300–400 nm) results in a reduction in the short-circuit current density (*J*_SC_) by approximately 2–5%, with corresponding PCE losses of up to 0.5–1.5 percentage points. This trade-off between efficiency and stability has become a key challenge in current research. In recent years, researchers have developed an innovative solution: employing photon conversion materials to transform harmful UV light into usable visible light [60,61]. Such materials primarily include rare-earth-doped down-conversion materials that convert UV photons into red light (612 nm); organic fluorescent molecules with large Stokes shifts; and quantum dot materials (e.g., CsPbBr_3_) capable of spectral tuning.

**Figure 3:**
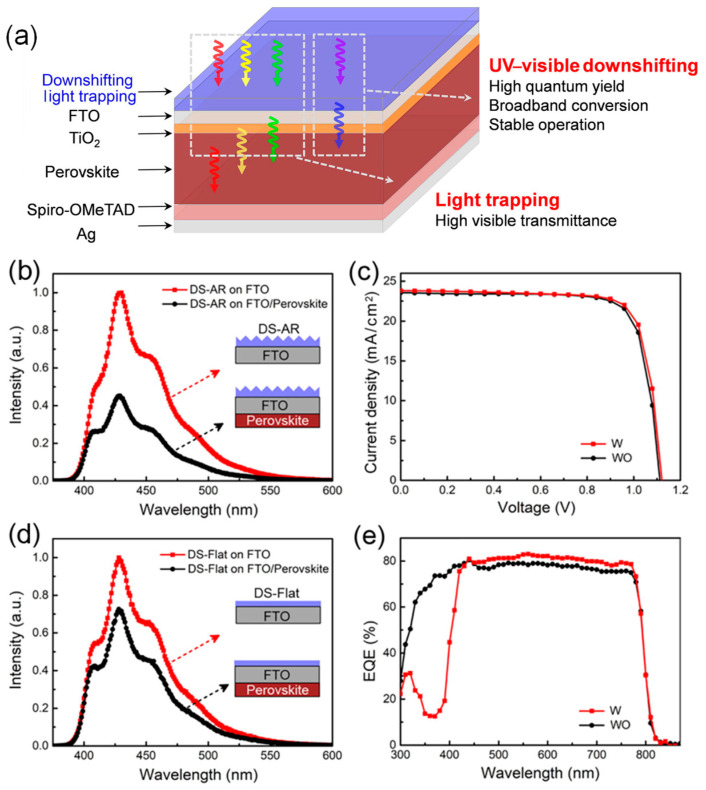
(**a**) Schematic drawing of perovskite solar cells with a spectral modification layer. (**b**) Photoluminescence (PL) spectra of DS-AR (down-shifting anti-reflection) coatings on FTO substrates with and without perovskite films. (**c**) *J*-*V* curves. (**d**) UV stability of PSCs with and without DS-AR layers. (**e**) External quantum efficiency (EQE) spectra. Reproduced with permission from [62], Copyright 2021, Elsevier.

Considering the spectral response characteristics of PSCs, Li et al. proposed introducing a spectral conversion layer on the surface of PSCs to modulate the spectrum. By integrating UV-to-visible spectral conversion and light-trapping functionalities within the conversion layer, they aimed to enhance UV stability without sacrificing PCE [62]. On one hand, the visible-light utilization was improved by fabricating an inverted pyramid pattern on a PDMS (polydimethylsiloxane) substrate using a templating method to reduce light reflection, thereby increasing visible-light transmittance by 5%. On the other hand, the detrimental effects of UV light were mitigated by incorporating the fluorescent molecule BBOT into the conversion layer. Upon UV excitation, BBOT emits photons within the visible range, which can generate photoexcited charge carriers in the perovskite layer. BBOT exhibits a strong broadband excitation spectrum from 250 to 400 nm and an emission spectrum covering 400 to 600 nm, with a peak at 450 nm. The emitted photon wavelengths are well-matched to the absorption range of the perovskite layer. As shown in Figure 3, compared to the control group without the spectral conversion layer, PSCs with the conversion layer exhibited lower current densities in the 300–400 nm range but higher currents in the 450–800 nm visible-light range. The experimental results demonstrated that the introduction of an optimized spectral conversion layer did not compromise the conversion efficiency while significantly improving UV stability. Importantly, the spectral conversion layer does not require any modification of the internal structure of PSCs, making it broadly applicable to various device architectures.

**Figure 4:**
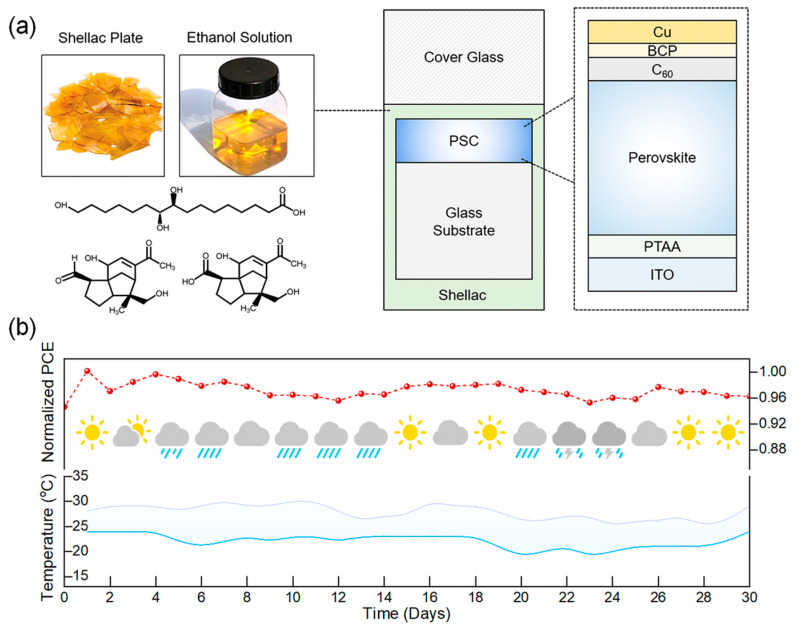
Shellac-based encapsulation process and outdoor stability of PSCs. (**a**) Schematic illustration of the shellac-based encapsulation process. PSCs are covered with a shellac film (20–50 μm thick) by spray-coating or drop-casting methods, followed by covering with a cover glass. (**b**) Outdoor stability of SE modules according to the MQT 08 (IEC 61215) protocol. Reproduced with permission from [63], Copyright 2024, Elsevier.

External encapsulation materials can effectively inhibit the permeation of environmental moisture and oxygen and attenuate UV irradiation by constructing physical barrier layers, thereby significantly enhancing the environmental stability of PSCs. Shao et al. employed shellac (SE) as a multifunctional encapsulation adhesive, offering multiple functions including moisture isolation, UV absorption, mechanical shock buffering, and lead leakage suppression (Figure 4) [63]. Moreover, the simple solution process and abundant raw materials substantially reduced the encapsulation cost. Ultimately, the encapsulated PSC modules passed outdoor stability tests, UV preconditioning tests, and hail impact tests according to the IEC 61215 standard [64].

The use of UV-shielding encapsulation layers in PSCs [65,66] results in the loss of ultraviolet photons from the solar spectrum, thereby limiting further improvements in efficiency. Therefore, a growing trend has emerged to design a layer that not only acts as an encapsulant, but also serves as a photon recycler for incident ultraviolet radiation. By incorporating luminescent down-shifting (LDS) fluorophores into the encapsulation layer, PSCs can be protected from environmental factors while converting high-energy photons into lower-energy photons [65,67,68,69,70]. These lower-energy photons can then be readily absorbed by the perovskite layer, and the increased number of absorbable visible photons subsequently enhances the external quantum efficiency (EQE) of PSCs.

**Figure 5:**
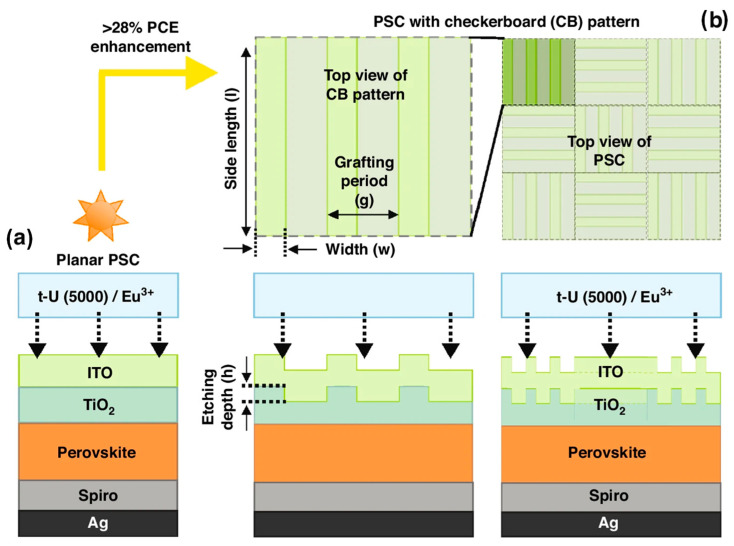
Sketch of the architecture of planar (**a**) and photonic-enhanced (**b**) PSCs with an LDS encapsulant coating composed of t-U (5000)/Eu^3+^. The innovative LT design applied on the front contact of the PSCs (**b**) consists of periodic grating lines that form a trellised checkerboard (CB) pattern in the TiO_2_ electron transport layer (ETL). The geometrical parameters (h, w, g, l) of the CB patterns (**b**) considered for optimization are indicated with arrows. Two distinct thicknesses (250 nm and 500 nm) were selected for the perovskite absorber layer. Reproduced with permission from [71], Copyright 2024, the author(s).

Mendes et al. [71] proposed a chessboard (CB) tiling pattern specifically designed for UV photon conversion (Figure 5). This photonic structure enhanced the photocurrent and PCE of ultrathin PSCs by 25.9% and 28.2%, respectively. They also identified a luminescent down-shifting encapsulant capable of converting UV irradiation into visible photons that match the absorption spectrum of the solar cells. The absorption and emission profiles of state-of-the-art down-shifting materials—specifically, lanthanide-based organic–inorganic hybrids—were experimentally obtained and used to predict the potential gains from UV energy utilization. It was demonstrated that at least 94% of incident UV radiation could be effectively converted into the visible spectral range. By integrating light harvesting with luminescent down-shifting layers, a potential photonic solution was revealed that could overcome UV-induced degradation in PSCs while mitigating optical losses in ultrathin cells, thereby improving both performance and stability.

### 3.2. Internal Strategies

Internal optimization strategies for the UV stability of PSCs can be systematically designed and controlled at different structural levels of the material system, primarily including three key aspects: perovskite bulk phase optimization, perovskite interface modification (mainly the buried interface), and charge transport layer optimization.

#### 3.2.1. Perovskite Bulk Phase

Variations in the type and content of elements in the perovskite bulk phase significantly affect the structural integrity and long-term stability of the perovskite crystals. Specifically, variations in the composition of A-site cations (e.g., Cs^+^, FA^+^, MA^+^) alter lattice parameters and octahedral tilting angles, thereby affecting the phase stability of the material. The choice of B-site metal ions (e.g., Pb^2+^, Sn^2+^) directly impacts the conduction band position and carrier mobility. Additionally, the ratio of X-site halide ions (I^−^, Br^−^, Cl^−^) is closely related to the bandgap width and photostability [72]. This composition–structure–performance correlation not only determines the intrinsic stability of perovskites, but also influences their degradation kinetics under environmental stresses such as light exposure and humidity. For example, the introduction of an appropriate amount of Cs^+^ can suppress phase separation, but excessive amounts can lead to lattice distortion [73]. Therefore, precise control over the type and content of elements in the perovskite bulk phase is a crucial foundation for achieving efficient and stable perovskite optoelectronic devices.

Reducing the Br content and increasing the Cs content lead to an increase in octahedral tilting and improved photostability. Stranks et al. found that an increase in the tilting of the perovskite octahedral structure enhances photostability, which is associated with a reduction in the density of hexagonal polymorphic phase impurities that accelerate photodegradation [74]. Studies show that composition acts as a lever to adjust crystal symmetry, thereby preventing the transition to hexagonal phase impurities and enhancing photostability. Lowering the Br content (larger average halide size) and/or increasing Cs content (smaller average A-site size) leads to increased octahedral tilting and a reduction in the number of hexagonal polymorphic phase impurities. This finding reveals the structural features that support photostability, and can therefore be used to make targeted modifications to halide perovskites, improving the commercial prospects of technologies based on these materials.

**Figure 6:**
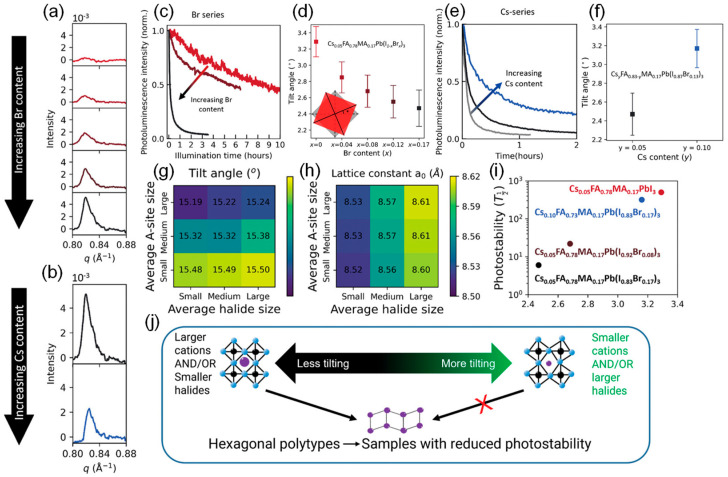
(**a**) Sample with composition Cs_0.05_FA_0.78_MA_0.17_Pb(I_1−x_Br_x_)_3_, where 0 ≤ x ≤ 0.17 (figure from top to bottom, Br content increases). (**b**) Samples with compositions Cs_0.05_FA_0.78_MA_0.17_Pb(I_0.83_Br_0.17_)_3_ (black) and Cs_0.10_FA_0.83_MA_0.17_Pb(I_0.83_Br_0.17_)_3_ (blue). (**c**,**e**) Integrated PL intensity spectra of perovskite films under oxygen-free sunlight exposure: (**c**) Cs_0.05_FA_0.78_MA_0.17_Pb(I_1−x_Br_x_)_3_, where x = 0 (bright red), x = 0.08 (dark red), and x = 0.17 (black); (**e**) Cs_y_FA_0.83−y_MA_0.17_Pb(I_0.83_Br_0.17_)_3_, where y = 0 (gray), y = 0.05 (black), and y = 0.10 (blue). (**d**,**f**) Relationship between octahedral tilting angles and Br; Cs content extracted from GIWAXS. The inset shows the tilting and reference non-tilted octahedra, with the octahedral tilting angles marked by *. (**g**,**h**) Heatmaps show the influence of halide size and A-site cation size on octahedral tilting angles and lattice constants (a_0_). (**i**) The scatter plot shows the relationship between photostability (time required for 50% PL intensity decay under light exposure) and octahedral tilting. (**j**) Schematic diagram. Reproduced with permission from [74], Copyright 2024, the author(s). *Advanced Materials*, published by Wiley-VCH GmbH.

Figure 6 illustrates the effects of Br and Cs content on photostability, octahedral tilting, and the formation of hexagonal polytypes, as well as the role of composition in modulating octahedral tilt angles and photostability. The presence of Br leads to decreased photostability [75]. The spectral integral intensities of the components with x = 0, x = 0.08, and x = 0.17 are plotted as a function of time. Br addition leads to an increase in the photodegradation rate over time under illumination. Br addition leads to the formation of more hexagonal polymorphic phases. The GIWAXS scattering intensity graphs integrate patterns displaying the Bragg peaks of the perovskite hexagonal polymorphic phase. Weak but detectable scattering intensities were observed in the range of 0.80 ≤ q ≤ 0.88 Å^−1^, corresponding to the hexagonal polymorphic phase with the space group P6_3_/mmc (2H to 6H), composed of face-shared [PbI_6_]^4-^ octahedra [76]. With the increase in Br content, an increase in scattering intensity within this range was observed. Similarly, Cs exhibits the opposite effect to Br.

The increase in ion conductivity in perovskite solar cells is significantly correlated with a decrease in photostability. This correlation is primarily reflected in the following aspects: High ion conductivity typically means that halide ions (I^−^, Br^−^) and cations (Pb^2+^, MA^+^) are more likely to migrate under illumination. This migration leads to phase separation and compositional segregation, increased defect state density, and the formation of non-radiative recombination centers [77]. Kanatzidis et al. discovered that incorporating enI_2_ (en = ethylenediamine) into the mixed halide composition Cs_0.2_FA_0.8_Pb(I_0.8_Br_0.2_)_3_ successfully led to the formation of hollow perovskite films [78]. The incorporation of this hollow perovskite structure significantly reduced the ion conductivity in the film and improved photostability compared to non-hollow perovskite samples. The hollow perovskite structure is formed by introducing diamines (e.g., ethylenediamine) as cations, whose size violates the geometric tolerance factor rule, leading to the expulsion of M and X atoms, creating metal and halide vacancies in the existing octahedral framework while retaining the overall 3D perovskite structure.

Introducing sunscreen additive molecules into perovskites is also an effective method for improving UV stability in perovskite solar cells. Song et al. synthesized a “sunscreen” molecule, 2-hydroxy-4-methoxybenzophenone (HOC_6_H_3_(OCH_3_)COC_6_H_5_) [79], which not only protects perovskite solar cells from UV degradation but also achieves molecular defect passivation through interactions between functional groups and molecular isomerization under UV exposure. Therefore, the sunscreen strategy effectively enhances the UV tolerance of PSCs and increases the defect formation energy to −1.35 eV. The sunscreen PSCs exhibited excellent efficiencies of up to 23.09% (0.04 cm^2^) and 19.73% (1.00 cm^2^), along with long-term UV (UVA: 365 nm; UVB: 285 nm) stability.

**Figure 7:**
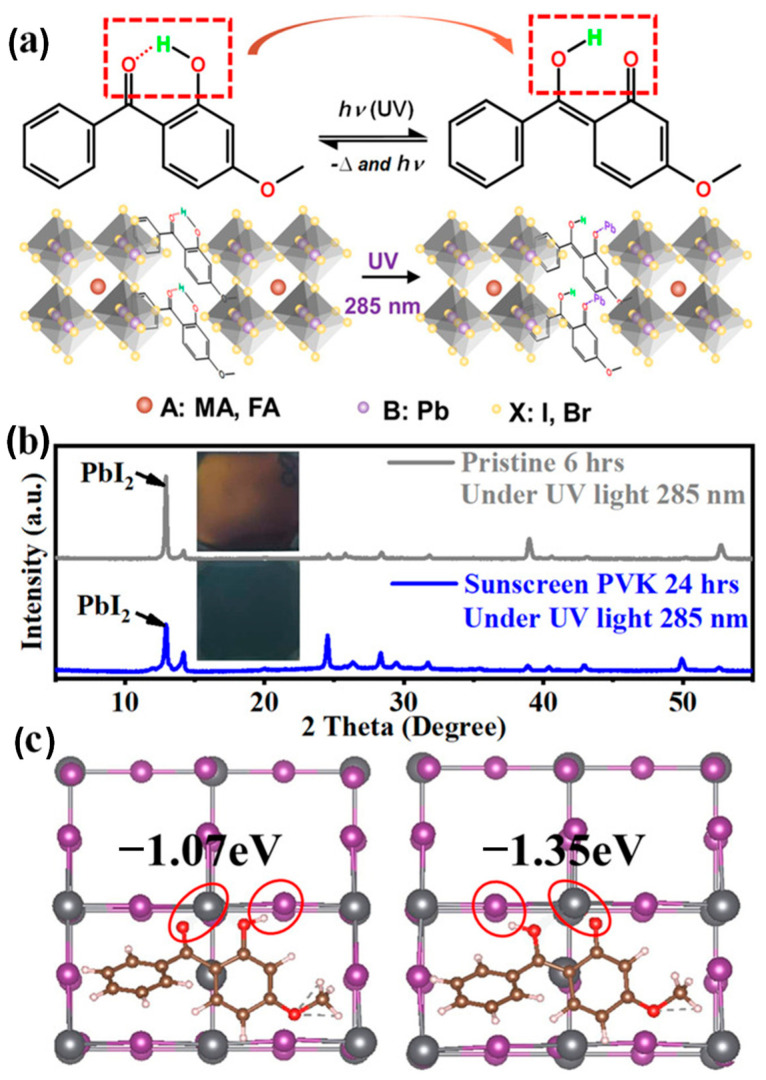
(**a**) Preparation and dynamic properties of sunscreen PSCs. (**b**) XRD spectra of perovskite films with and without “sunscreen” under UVB 285 nm illumination. (**c**) DFT calculations of the sunscreen isomeric molecules for Pb antibody surface passivation. Reproduced with permission from [79], Copyright 2021, Wiley-VCH GmbH.

Figure 7a illustrates the sunscreen and passivation mechanisms of perovskites: 2-hydroxy-4-methoxybenzophenone (HOC_6_H_3_(OCH_3_)COC_6_H_5_) acts as a UV absorber, containing a hydrogen atom in the hydroxyl group and oxygen in the adjacent carbonyl group, which can form intramolecular hydrogen bonds and chelated rings within the biphenyl ketone structure. Under UV exposure, the hydrogen bonds break, and the chelated rings open. UVA and UVB light are consumed, and an enol structure is formed, enabling both sunscreen and passivation capabilities. Thus, the sunscreen perovskite survives UV- and defect-induced degradation through interactions between the carbonyl group of the isomerizing molecules and the defects. Figure 7b presents photos and XRD spectra of perovskite films with and without sunscreen under UVB 285 nm illumination, confirming the protective effect of the sunscreen molecules. After 6 h of UVB exposure, the perovskite film turned yellow, and the PbI_2_ peak at 12.6° showed a significant increase. In contrast, the sunscreen perovskite film maintained its normal black color and standard perovskite diffraction peaks after 24 h of UV 285 nm exposure. These results clearly demonstrate the protective effect of sunscreen.

#### 3.2.2. Perovskite Buried Interface

Optimizing the buried interface quality or modifying it can significantly enhance the photostability of PSCs. Zhao et al. demonstrated a strategy to enhance the buried interface with CsI, achieving both high efficiency and UV-resistant perovskite solar cells [80]. High-performance PSCs based on FAPbI_3_ were fabricated using CsI-SnO_2_ composite ETLs, exhibiting improved PCE and UV stability. This chemical incorporation of CsI with SnO_2_ induces the vertical growth of perovskite grains, significantly improving crystallinity while maintaining the intrinsic FAPbI_3_ composition. Additionally, the abundance of CsI at the SnO_2_/perovskite interface helps reduce defects and achieve better band alignment, thereby alleviating carrier recombination and accelerating charge transport. As a result, the photovoltaic performance of the device was enhanced, with the CsI-modified device achieving an efficiency of 23.3%. At the same time, the CsI-modified devices maintained up to 88% of their initial efficiency after 500 h of UV exposure, with negligible degradation of the FAPbI_3_ perovskite.

**Figure 8:**
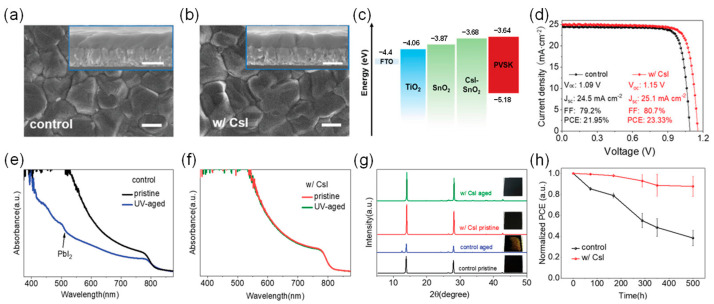
(**a**) Top-view and cross-sectional (inset) SEM images of perovskite films on SnO_2_ and (**b**) CsI-SnO_2_. The scale bar represents 500 nm. (**c**) Schematic energy level diagram. (**d**) *J*-*V* curve of the best-performing PSC measured. (**e**) UV–visible absorption spectra of original and UV-aged perovskite films based on SnO_2_ and (**f**) CsI-SnO_2_ ETLs. (**g**) XRD spectra and corresponding photos of original and UV-aged perovskite films based on SnO_2_ and CsI-SnO_2_ ETLs. (**h**) Normalized PCE decay curves of SnO_2_- and CsI-SnO_2_-based PSCs under 365 nm UV exposure. Aging tests were conducted in a N_2_ glovebox. Reproduced with permission from [80], Copyright 2022, Wiley-VCH GmbH.

UV aging tests of perovskite films and devices were conducted for 500 h under 365 nm UV light with an illumination intensity of 36.4 mW·cm^−2^. The UV intensity applied here is approximately eight times that of AM1.5 solar light (about 4.6 mW·cm^−2^ UV). As shown in Figure 8, after UV exposure, the absorbance of the bare SnO_2_-based perovskite film significantly decreased, and a PbI_2_ absorption edge appeared at 518 nm, indicating partial degradation of the film. In contrast, the CsI-SnO_2_-based film showed little change in its absorption spectrum. After UV aging for 500 h, the XRD peak intensities of the bare SnO_2_-based film at 14.1° and 28.2° significantly decreased, and a PbI_2_ peak appeared at 12.6°, with the film turning partially yellow, confirming UV-induced degradation. In contrast, the CsI-SnO_2_-based film’s XRD peaks remained stable, and its color remained black, demonstrating excellent UV stability. Device testing showed that the CsI-SnO_2_-based devices retained 88% of their initial PCE after 500 h of UV exposure, while the bare SnO_2_-based devices retained only 38%.

**Figure 9:**
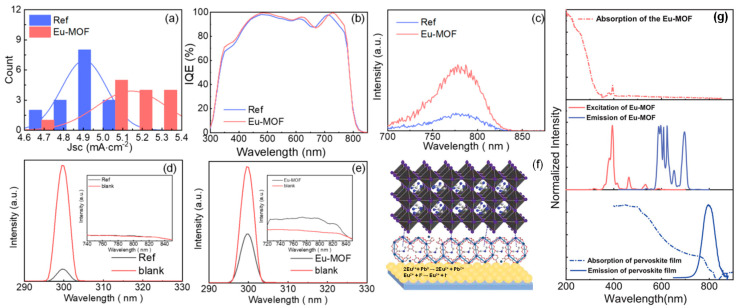
(**a**) Efficiency histogram of integrated photocurrent density in the 300–500 nm range. (**b**) IQE spectra. (**c**) Emission PL spectra under 250 nm excitation. Perovskite films: (**d**) without Eu-MOF and (**e**) with Eu-MOF, for PLQE measurements under 300 nm excitation. (**f**) Schematic diagram of the effect of Eu-MOF on perovskite films. (**g**) Spectra of different compositions: Eu-MOF UV absorption spectrum (top), Eu-MOF excitation spectrum (middle, red curve), and emission spectrum (middle, blue curve); FAMAC perovskite film absorption (bottom, dashed line) and emission (bottom, solid line) spectra. Reproduced with permission from [81], Copyright 2021, Wiley-VCH GmbH.

Other researchers have applied a Eu-MOF interlayer at the bottom of the perovskite crystal as a UV filter to protect the solar cell. Chen et al. designed a Eu-MOF interlayer at the bottom of the perovskite crystal, which enhances light utilization through down-conversion, broadens the light absorption range, reduces perovskite degradation, and improves device efficiency and stability (Figure 9) [81]. Through integrating photocurrent density and IQE tests, it was found that the performance of the Eu-MOF device outperformed the control group in the 300–500 nm wavelength range. Additionally, photoluminescence quantum efficiency (PLQE) tests showed that the Eu-MOF treatment improved the film’s PLQE from 0% to 0.58%. In the PL spectrum, Eu-MOF shows an excitation peak at 393 nm and an emission peak in the 570–720 nm wavelength range. Notably, the excitation peak of Eu-MOF overlaps well with the perovskite absorption band, providing the necessary conditions for efficient Förster resonance energy transfer (FRET) between the two. This energy transfer mechanism allows high-energy UV photons to be first absorbed by the Eu-MOF layer, emitting photons in the visible range through the down-conversion process, which are then effectively utilized by the perovskite layer, significantly enhancing the device’s light energy utilization. These results demonstrate that Eu-MOF absorbs UV light and down-converts it to emit visible light in the 300–500 nm range, which not only effectively suppresses UV-induced degradation, but also significantly increases *J*_SC_.

**Figure 10:**
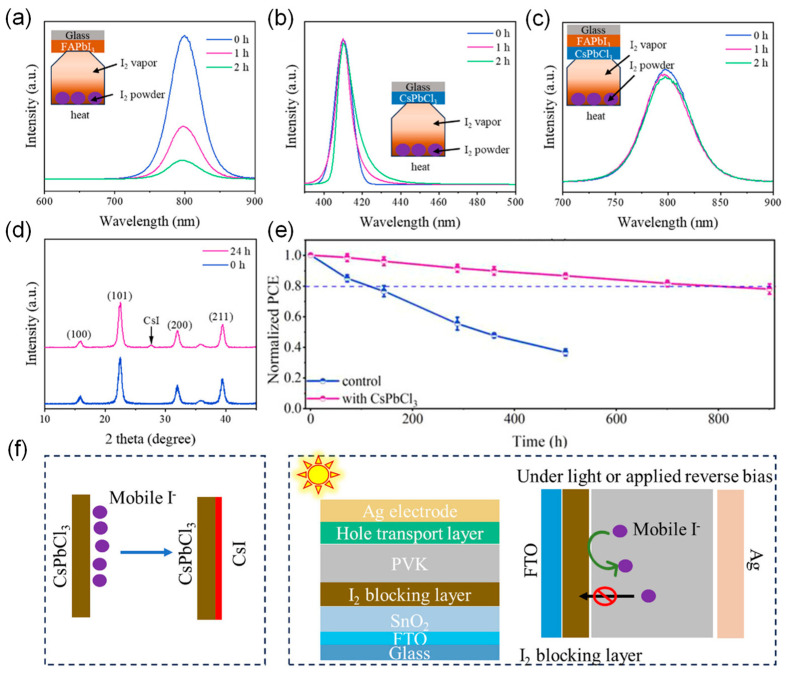
(**a**,**b**) PL spectra of FAPbI_3_ and CsPbCl_3_ films under I_2_ vapor exposure for different times. Inset: Schematic diagram of I_2_ vapor exposure to perovskite films in the dark and in a nitrogen-filled glovebox. (**c**) PL spectra of CsPbCl_3_/FAPbI_3_ under I_2_ vapor exposure for different times. (**d**) XRD spectra of CsPbCl_3_ perovskite after more than 24 h of I_2_ vapor exposure. (**e**) Normalized PCE of unencapsulated PSCs under 365 nm UV exposure. (**f**) Mechanism of CsPbCl_3_ used as an I_2_ blocking layer. Reproduced with permission from [86], Copyright 2025, Wiley-VCH GmbH.

Perovskite nanocrystals (NCs) with similar composition and lattice structure to the perovskite layer can act as seeds for the uneven crystallization and growth of the film, reducing both buried-interfacial and bulk defects, increasing ion migration activation energy, and minimizing ion migration [82], thereby enhancing the stability and efficiency of perovskite devices [83,84,85]. Chen et al. innovatively used CsPbCl_3_ nanocrystals (NCs) as a buried interface blocking layer, significantly enhancing the efficiency and photostability of PSCs [86]. Studies show that the CsPbCl_3_ NC interface layer operates through the following synergistic mechanisms: (i) spatial confinement effectively inhibits iodide ion migration; (ii) a reaction occurs with diffusing iodide ions to form a CsI barrier film, preventing iodine leakage. These mechanisms lead to a significant improvement in device performance, with PCE increasing from 22.06% to 24.66%, demonstrating excellent operational stability. Under UV exposure, the T_80_ lifetime was extended by 8 times, and the T_80_ lifetime increased by 7 times under combined thermal and light-aging tests (Figure 10).

#### 3.2.3. Charge Transport Layers

Currently, organic HTLs are widely used in high-performance inverted PSCs due to their excellent hole-extraction capability. In particular, self-assembled monolayers (SAMs), such as the carbazole-based PACz series, have been reported to achieve power conversion efficiencies exceeding 25% [87,88].

**Figure 11:**
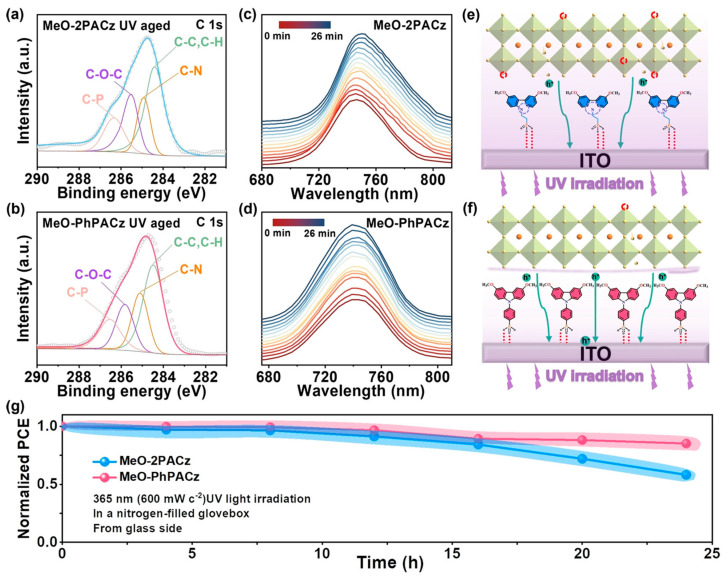
Effect of MeO-2PACz and MeO-PhPACz on device UV stability. (**a**,**b**) XPS spectra of the C1s region after 24 h of 365 nm UV exposure, with each peak representing the ratio of atomic types to the total number of atoms in the molecular structure. (**c**) In situ time-dependent PL spectra of MeO-2PACz- and (**d**) MeO-PhPACz-based perovskite films under 20 min of continuous CW 405 nm laser exposure in a N_2_ atmosphere. (**e**,**f**) Schematic diagrams of SAMs as HSLs interacting with ITO and perovskite. (**g**) UV stability measurement of PSCs under 365 nm UV exposure (600 mW·cm^−2^) in a N_2_-filled glovebox. Reproduced with permission from [89], Copyright 2023, Wiley-VCH GmbH.

Gao et al. synthesized a novel SAM compound, MeO-PhPACz, based on fully aromatic carbazole and found that MeO-PhPACz exhibits intrinsic UV resistance and enhanced UV absorption, significantly improving the UV resistance of the target PSCs [89]. The performance of MeO-PhPACz as a hole-selective layer (HSL) in an inverted wide-bandgap (1.68 eV) PSC was studied and compared with MeO-2PACz, resulting in an optimized device with a PCE of 21.10%, compared to 19.53% for MeO-2PACz. More importantly, the intrinsic UV resistance and stronger UV absorption capability of the MeO-PhPACz structure make the target PSC exhibit significantly higher UV resistance compared to the MeO-2PACz-based PSC. The use of conjugated linkers stabilizes the electron-rich carbazole part through efficient electron/charge delocalization, resulting in a significant increase in the substrate’s dipole moment and modulated work function (WF). MeO-PhPACz exhibits a larger dipole moment, stronger hole extraction kinetics, and better hole transport properties, making it a more effective hole extraction channel. Additionally, the aromatic linkers provide sufficient orbital overlap to induce conjugation, thereby reducing interfacial non-radiative recombination. As shown in Figure 11, after UV exposure (365 nm), the C-N content of MeO-2PACz/ITO decreased from 24.41% to 13.61%, while MeO-PhPACz/ITO remained stable, demonstrating excellent UV resistance. The MeO-2PACz-based perovskite film exhibits a PL peak redshift and broadening after laser aging, which are typical characteristics of phase segregation, indicating phase separation. In contrast, the MeO-PhPACz-based film’s PL peak remains unchanged, effectively suppressing photo-induced phase segregation. Under 365 nm UV exposure, the PSC with MeO-PhPACz retains 85% of its initial PCE, outperforming MeO-2PACz (which retains < 60%). The MeO-PhPACz hole transport layer absorbs UV light, significantly slowing the decay of PCE under UV exposure from the glass side, which has significant implications for photovoltaic development.

However, organic HTLs lack long-term stability under high temperatures and/or high-energy UV illumination, which could be a significant obstacle for practical applications [90]. Additionally, the high cost of organic materials is unfavorable for future commercialization [91]. Therefore, significant efforts have been made to develop intrinsically stable and low-cost inorganic hole transport materials, including CuSCN [92], NiO_x_ [93], CuI [94], and others. Among these, metal oxides exhibit the greatest potential due to their high transparency, high conductivity, and simple fabrication. Although the metal vacancy defects commonly present on the film surface are a challenging issue, they significantly reduce the efficiency and stability of PSCs.

**Figure 12:**
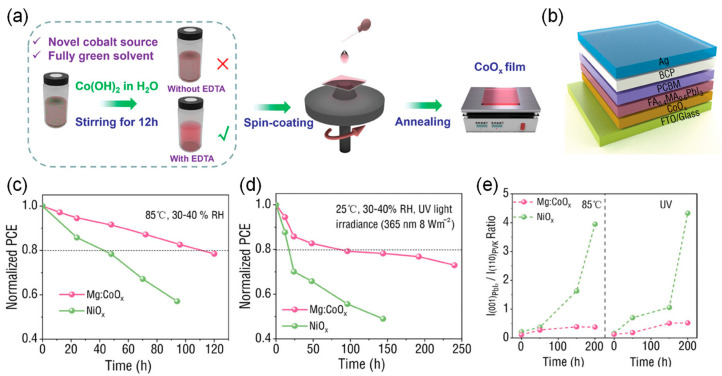
(**a**) Schematic diagram of the solution processing method used for CoO_x_ preparation. (**b**) Schematic diagram of a complete PSC. (**c**,**d**) Thermal stability and UV stability of PSCs based on Mg:CoO_x_ and NiO_x_ HTLs under 85 °C aging and continuous UV exposure (365 nm, 8 W·m^−2^) with approximately 40% relative humidity. (**e**) Change in the ratio of peak intensities of (001) PbI_2_/(110) perovskite (PVSK) in perovskite films based on Mg:CoO_x_ and NiO_x_ HTLs under 85 °C aging and continuous UV exposure (365 nm, 8 W·m^−2^) with approximately 40% RH. Reproduced with permission from [95], Copyright 2025, Wiley-VCH GmbH.

Tai et al. first used Co(OH)_2_ as a cobalt source and water as an eco-friendly solvent to fabricate cobalt oxide (CoO_x_) HTLs with defect-free surfaces through a solid solution process (Figure 12) [95]. Compared to traditional NiO_x_ HTLs, PSCs based on CoO_x_ HTLs exhibit excellent thermal stability and UV stability. Theoretical calculations show that CoO_x_ has a higher metal vacancy defect formation energy and higher interface adhesion energy than NiO_x_, leading to a chemically stable HTL/perovskite interface. After further processing the microstructure and electronic properties of CoO_x_ HTLs by doping with magnesium acetate (MgAc_2_), the efficiency of the FA_0.4_MA_0.6_PbI_3_ light-absorbing layer processed at room temperature reached 22.35%, surpassing all reported results for CoO_x_-based PSCs, and a higher value of >24% could be achieved through simple interface modifications. The corresponding devices also demonstrated robust operational stability in air without the need for encapsulation.

## 4. Conclusions and Outlook

### 4.1. Conclusions

Perovskite solar cells have become a research hotspot in the photovoltaic field due to their high efficiency (>26%), low cost, and solution processability [96,97,98]. However, their commercialization process is still hindered by long-term stability issues, with photostability and ultraviolet stability being particularly important concerns. This review first analyzes the factors affecting the UV stability of PSCs and the mechanisms of UV degradation. The issues of photostability/UV stability in PSCs mainly stem from intrinsic material degradation (such as halide segregation and ion migration) and interfacial failures (such as UV-induced oxidation and defect accumulation). Subsequently, this review provides a general summary of external and internal solutions, including the introduction of external UV blocking layers, encapsulation strategies, bulk doping, compositional tuning, and the contributions of charge transport layers (CTLs). The aim is to provide some guidance in this field and give readers a preliminary understanding of the photostability issues in perovskite solar cells. Table 1 summarizes the external and internal strategies for improving the UV stability of perovskite solar cells.

### 4.2. Outlook

While progress has been made in improving the UV stability of PSCs through external strategies, several key issues still need special attention: The first issue is spectral loss. When adopting overly aggressive UV cutoff strategies (such as setting the cutoff wavelength below 400 nm), although UV light is effectively blocked, some blue light spectrum is inevitably lost, leading to a significant decrease in *J*_SC_ by approximately 2–5%, which has a considerable impact on the overall power conversion efficiency of the device [99]. The second challenge is thermal management. Studies have shown that for every 1% increase in UV light blocking, the operating temperature of the module increases by 0.3–0.5 °C. The UV light energy that is blocked does not completely disappear, but rather transforms into heat, accumulating within the device. This thermal accumulation not only affects device performance, but may also accelerate the aging and degradation of encapsulation materials [99]. In terms of internal modification strategies, researchers face more complex challenges. Although existing interface modification layers (such as Al_2_O_3_, MgO, etc.) can partially suppress UV-induced interfacial degradation, these modification materials often introduce additional carrier recombination centers at the interface or increase the series resistance of the device, negatively affecting charge transport efficiency [100]. Ion doping (such as introducing Rb^+^, Cs^+^, etc.) or constructing low-dimensional perovskite structures (such as 2D/3D heterojunctions) can effectively suppress harmful ion migration, but these improvements often come at the cost of reduced charge transport efficiency or significantly increase the complexity of the fabrication process, making them less favorable for industrial applications [101]. In terms of material compositional tuning, widening the bandgap by increasing the Br content (e.g., preparing MAPb(I_1−x_Br_x_)_3_ solid solutions) can reduce UV light absorption, but this approach is prone to photo-induced halogen segregation, leading to phase separation issues [102]. Currently, the scientific community still lacks a universal compositional design guideline to balance bandgap tuning with phase stability.

Additionally, the application of UV absorbers or down-conversion materials also has obvious limitations. While these functional materials can shield harmful UV radiation, they often suffer from poor photostability or incompatibility with the perovskite active layer, leading to failure in practical applications [45,103]. Defect passivation strategies also face challenges. Common passivators (such as thiol compounds, specific polymers, etc.) can effectively reduce non-radiative recombination in the initial stages, but under prolonged UV exposure, they may undergo photochemical reactions (such as C-S bond cleavage), which can accelerate the degradation of device performance [104]. More complex is the fact that in real-world application environments, UV aging often interacts synergistically with other environmental factors, such as humidity and temperature, leading to multiple degradation pathways occurring simultaneously (e.g., UV–humidity–thermal synergistic degradation effects). However, most current studies are still limited to testing under single-stress conditions and lack a deep understanding of the coupling of multiple factors [10]. Therefore, there is an urgent need to establish accelerated aging testing protocols and evaluation systems that more closely mimic real-world application environments.

Despite significant improvements in stability, the 20-year lifespan required for commercialization is still not met, and the technology does not yet satisfy the demands of commercial applications. As the saying goes, “A device that survives 1000 h in the lab is still a whole ocean away from meeting the 20-year outdoor power generation requirement”. To further increase stability, additional technologies and new application environments should be considered in conjunction with other strategies [105]. Compared to outdoor conditions, indoor light and heat are milder. Therefore, the degradation mechanisms of indoor photovoltaics differ from those of outdoor photovoltaics. Mainly, a small number of photoelectrons are generated, allowing the partial filling of trap states, and the UV impact is generally not considered indoors, thereby slowing down degradation [106]. Due to the fewer photons received by the device, the output power generated by indoor photovoltaics is lower [106]. At this stage, the PCE of Cs_0.17_FA_0.83_PbI_x_Br_3−x_ indoor photovoltaics has reached 36.36% [107]. By reducing the defect density of the perovskite through composite and interface engineering to suppress leakage current and optimizing the perovskite composition to match the visible-light emission spectrum of indoor light sources, a significant increase in PCE can be achieved. Min et al. [108] attempted to use flexible quasi-2D perovskite solar cell modules to power wearable biosensors, providing continuous and non-invasive metabolic monitoring. The flexible device provides sufficient power in both outdoor and indoor conditions (with a PCE exceeding 31% under indoor illumination), allowing the biosensor to operate continuously for 12 h. This work confirms the feasibility of integrating PSCs with multiple indoor devices. Moreover, PSCs have demonstrated tremendous potential for space applications [109,110]. Compared with terrestrial environments, the extreme conditions in space offer unique advantages while also posing new challenges. Firstly, the near-vacuum conditions in space effectively eliminate the presence of moisture and oxygen, fundamentally addressing one of the primary degradation mechanisms faced by PSCs in terrestrial applications. However, the intense UV radiation in space—10 to 100 times stronger than on Earth—imposes much stricter requirements on device stability. To advance PSCs for space deployment, comprehensive research is required across multiple dimensions: (1) Developing novel UV-protective encapsulation materials that combine high optical transparency (>90%) with either excellent UV blocking capacity (blocking rate >99% below 400 nm) or effective UV light conversion capability. We propose an innovative strategy: incorporating UV-converting materials into the encapsulation layer. This approach is both promising and feasible, as it does not discard the UV photons harmful to perovskites, but rather converts them into wavelengths that are usable and harmless to the perovskite absorber. Therefore, key research questions include how to design the molecular structure of such materials, extend their long-term stability, and achieve better integration with encapsulation systems. This strategy, which does not alter the internal structure of the device, undoubtedly improves the reproducibility of device performance. (2) Optimizing the perovskite material system to enhance intrinsic stability. (3) Innovating device architectures, such as by developing UV-reflective electrodes and designing graded energy-level structures. (4) Introducing functional additives—such as UV absorbers, converters, and radical scavengers—to construct multi-layered protection mechanisms. (5) Another promising approach is embedding metal nanoparticles into the perovskite layer to form plasmonic hubs, thereby enhancing photon absorption and compensating for efficiency losses caused by UV filtering [111]. Breakthroughs in these key technologies will greatly enhance the adaptability of PSCs to the space environment, offering efficient and reliable energy conversion solutions for future spacecraft such as space stations and deep-space probes. This represents an important scientific challenge that urgently awaits resolution by researchers in the field.

In summary, we firmly believe that breakthroughs in key technological pathways—selecting and developing perovskite material systems with stable optically active phases, precisely controlling defect formation mechanisms to reduce defect density, designing compatible interface modification strategies, optimizing the energy level alignment of charge transport layers, and developing highly reliable encapsulation technologies—will gradually overcome the optical instability challenges of perovskite materials, enabling them to withstand the harsh environmental tests of various complex application scenarios. In the foreseeable future, perovskite photovoltaic technology, with its unique commercialization advantages of low-cost fabrication, high photoelectric conversion efficiency, and flexible scalability, is expected to become the core breakthrough direction for the next generation of energy harvesting technologies, providing global users with more efficient and sustainable clean energy solutions.

## Figures and Tables

**Table 1:** Summary of strategies for enhancing UV stability in perovskite solar cells.

Category	Materials	Groups	PCE (%)	UV Stability *	Test Conditions	Ref.
Encapsulation	FTH Paper	Target	20.26	81%	100 h under 253 nm UV lamp, 384.61 mW/cm^2^	[59]
		Control	20.80	55%		
		Contrast	−0.54	+26%		
	BBOT	Target	21.10	95%	720 h under 365 nm UV lamp, 5 mW/cm^2^	[62]
		Control	20.70	60%		
		Contrast	+0.40	+35%		
	Shellac plate	Target	Not mentioned	91%	280 h under 253 nm UV light and heated to 358 K. 15 kWh/m^2^	[63]
		Control		69%		
		Contrast		+22%		
Bulk phase	Sunscreen	Target	23.09	80%	24 h under 285 nm, 1.35 mW/cm^2^, 6 h under 365 nm, 600 mW/cm^2^	[79]
		Control	19.82	20%		
		Contrast	+3.27	+60%		
Buried interface	CsI-SnO_2_	Target	23.33	88%	500 h under 365 nm UV lamp, 36.4 mW/cm^2^	[80]
		Control	21.95	38%		
		Contrast	+1.38	+50%		
	Eu-MOF	Target	22.16	85%	1000 h light with a white LED lamp, at 0.94 V and 1 sun illumination	[81]
		Control	19.73	75%		
		Contrast	+2.43	+10%		
	CsPbCl_3_ NCs	Target	24.66	80%	800 h under 365 nm UV lamp	[86]
		Control	22.06	40%		
		Contrast	+2.60	+40%		
CTLs	MeO-PhPACz	Target	21.10	85%	24 h under 365 nm UV lamp, 600 mW/cm^2^	[89]
		Control	19.53	60%		
		Contrast	+1.57	+25%		
	Mg:CoOx	Target	22.35	80%	150 h under 365 nm UV lamp, 8 W/m^2^	[95]
		Control	19.46	50%		
		Contrast	+2.89	+30%		

* UV stability is evaluated based on the percentage of PCE retained compared to the initial value.

## Data Availability

No new data were created or analyzed in this study.
